# Outcomes after inguinal hernia repair: how strong is the evidence for minimally invasive versus open repair?

**DOI:** 10.1007/s10029-026-03824-2

**Published:** 2026-09-05

**Authors:** Benjamin A. Y. Cher, Qinyun Lin, Kenneth A. Frank, Courtney J. Balentine

**Affiliations:** 1 Wisconsin Surgical Outcomes Research Program (WiSOR), Department of General Surgery, School of Medicine and Public Health, University of Wisconsin-Madison, 600 Highland Ave, Madison, WI 53792 USA; 2 School of Public Health and Community Medicine, University of Gothenburg, Box 100, 405 30 Gothenburg, Sweden; 3 College of Education, Michigan State University, 620 Farm Ln #134, East Lansing, MI 48824 USA

**Keywords:** Inguinal hernia, Minimally invasive surgery, Laparoscopic surgery, Open surgery, Hernia recurrence, Chronic pain, Robustness of inferences to replacement

## Abstract

**Purpose:**

It remains controversial whether minimally invasive surgery (MIS) is superior to open surgery for inguinal hernia repair. MIS is growing in popularity, but open surgery remains more common. Consensus remains elusive even though numerous studies have been published about the topic. A new method for sensitivity analysis, the Robustness of Inferences to Replacement (RIR), can summarize the complex body of literature and evaluate the strength of the published findings to better support surgical decision-making. This study applied the RIR to the available evidence about this topic.

**Methods:**

We reviewed studies from published meta-analyses that compared MIS versus open surgery for elective, unilateral inguinal hernia repair and calculated the RIR for each study and for the cumulative data. The RIR indicates how many patients in a published study must be replaced from a null-hypothesis population for the published result to be overturned. Higher RIRs indicate more robust conclusions.

**Results:**

The literature review identified 40 randomized trials for inclusion. In the cumulative analysis, MIS had higher incidence of hernia recurrence (RIR 21), lower incidence of chronic pain (RIR 276), and lower immediate post-operative pain (RIR 1,282).

**Conclusion:**

The evidence supporting MIS for decreased post-operative pain and chronic pain is substantially stronger than the evidence supporting open surgery for decreased hernia recurrence. However, there is likely not a clearly superior method for all patients. The findings demonstrate how the RIR can support better surgical decision-making by helping surgeons to interpret a large and complex body of published literature.

**Supplementary Information:**

The online version contains supplementary material available at https://doi.org/10.1007/s10029-026-03824-2.

## Introduction

Debates about new surgical techniques often devolve into irreconcilable statements of conflicting opinions rather than being objective assessments of the strength of available evidence [1]. One reason this occurs is because surgeons lack tools to quantify the relative strengths of conclusions from different studies as a basis for making decisions [2]. As a result, for many critical questions in surgery, consensus remains elusive even though numerous research studies have been published. It is often difficult to know whether evidence from additional studies would be strong enough to drive practice change. One important example is the debate about minimally invasive surgery (MIS) versus open surgery for elective unilateral inguinal hernia repair. Advocates of open surgery argue that MIS leads to more recurrences and that any advantages related to pain control are of minimal clinical significance [3, 4], while supporters of MIS counter that technical improvements and more experience have reduced the risks of hernia recurrence and other complications [5–8]. There is still no consensus about the best approach to repair.

Recently, methodologists have developed a novel method called the Robustness of Inference to Replacement (RIR), which can help resolve complex debates about the relative strengths and weaknesses of study results [2, 9, 10]. The RIR quantifies the robustness of study findings in terms of how resistant results are to hypothetical changes in patient outcomes. Unlike p-values and confidence intervals, the RIR has a more straightforward interpretation in terms of actual patient experiences, which supports its use for clinical decision-making [11]. The RIR can compare robustness across multiple outcomes within one study and the same outcome across multiple studies, and can be applied to cumulative meta-analyses to assess how the strength of evidence changes over time and whether additional studies are likely to yield informative new results.

In this study, we applied the RIR to the debate about MIS versus open surgery for elective unilateral inguinal hernia repair, measuring the strength of all available evidence for several relevant post-operative outcomes. The purpose was to help surgeons develop a better understanding of the entirety of the available evidence to make more informed decisions about surgical approach. The study also demonstrates how the RIR can enhance surgical decision-making by interpreting large numbers of studies with sometimes contradictory results.

## Methods

### Literature review

**Fig. 1:**
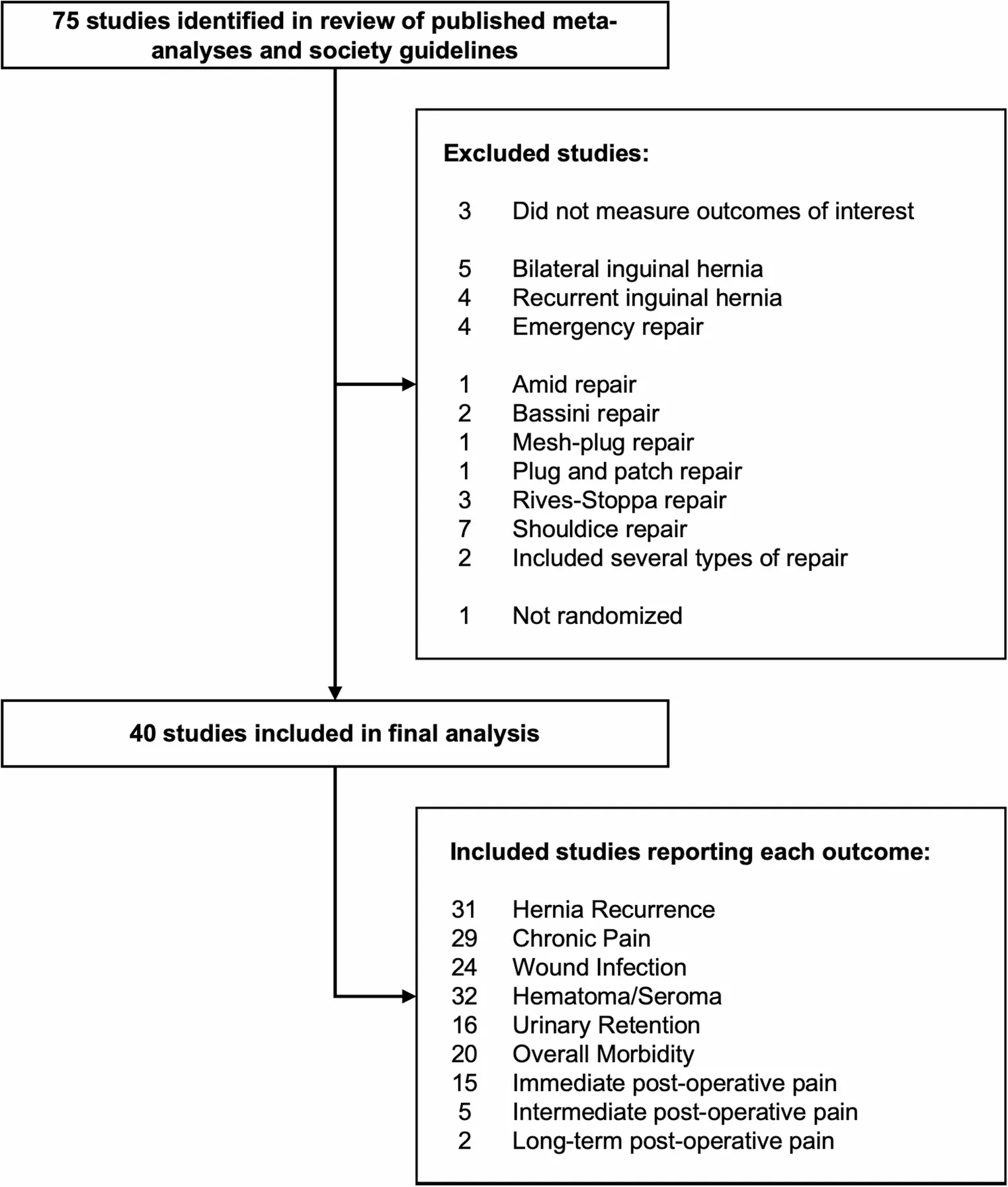
Diagram of literature search and study inclusion

We reviewed published meta-analyses to identify randomized trials comparing open versus MIS approaches to unilateral elective inguinal hernia repair (Fig. 1) [6, 7]. We did not seek to replicate the published meta-analyses nor conduct a new meta-analysis, but rather to demonstrate how the RIR can be used to better understand the robustness of already-published findings. 

We excluded studies of recurrent or bilateral inguinal hernias and emergent repairs. For open surgery, we limited the analysis to Lichtenstein’s tension-free repair. For MIS, we did not distinguish between laparoscopic and robotic approaches, and we included both trans-abdominal pre-peritoneal (TAPP) and totally extra-peritoneal (TEP) repair. When a study included both, TAPP and TEP, we added the numbers together. There was no cut-off for publication year, so the cumulative sensitivity analysis would reflect all available evidence since MIS was first developed.

### Outcomes

Long-term outcomes included hernia recurrence and self-reported presence/absence of chronic pain. Short-term post-operative outcomes included overall morbidity, immediate post-operative pain (visual analog scale [VAS]), wound infection, and urinary retention. We also included intermediate (1–2 weeks) and long-term (1–2 months) post-operative pain on the VAS.

### Overview of the RIR

**Fig. 2:**
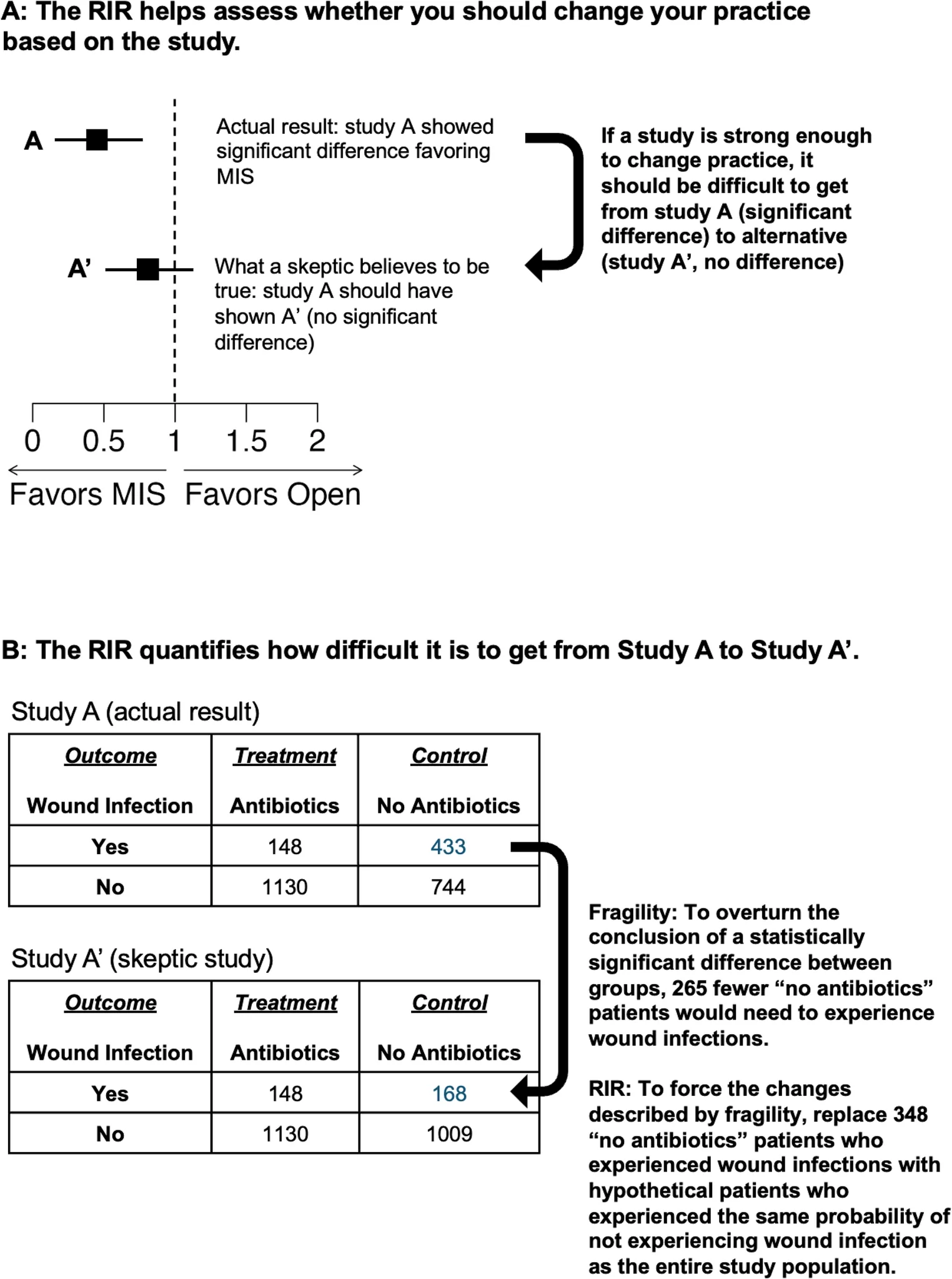
Conceptual overview of the robustness of inference to replacement (RIR)

Figure 2 A depicts a hypothetical study (Study A) that found a statistically significant difference favoring MIS over open surgery. A surgeon skeptical of Study A may believe the result was a mistake and that a new study would find no significant difference. The RIR describes what that study would look like; specifically, the smallest difference from Study A that would change the result so there is no longer any advantage to MIS (Study A’). If Study A is radically different from Study A’, the strength of Study A is greater and should support a change in practice.

RIR calculation involves two steps: (1) calculating Fragility, and (2) converting Fragility into the RIR. Fragility represents the absolute number of patients who would need to experience a different outcome for study results to change [12]. In Fig. 2B, Study A depicts real data from a Cochrane systematic review of prophylactic preoperative antibiotics and rates of postoperative wound infection after colorectal surgery [13], an example chosen because most surgeons accept that preoperative antibiotics reduce infections, making them standard of care. In Study A (actual results), antibiotics decreased wound infections (148/1,278 [12%] versus 433/1,610 [27%], *p* < 0.05). A Fragility value of 265 means that for there to be no significant advantage to antibiotics in a hypothetical new trial, the number of infections in the no-antibiotic group would need to fall from 433 to 168. A surgeon skeptic could then ask how likely it would be for a new trial to show such a substantial difference and use that to evaluate the strength of the original findings.

Fragility has been used in other sensitivity analyses [12] but has an important limitation: it depends heavily on how frequently the outcome occurs, making it difficult to compare across studies or across outcomes with different frequencies. This demonstrates the importance of converting Fragility into the RIR. To control for outcome frequency, Fragility is divided by the rate of “success” (no wound infection) in the entire study population. This yields the RIR, which measures how difficult it would be to generate the changes described by Fragility by replacing real patients with hypothetical patients from a null hypothesis population where antibiotics have no impact on wound infection. In this example, the RIR is 348, equal to 265 (Fragility) divided by the probability of no wound infection across both groups ([744 + 1,130]/[433 + 148+744 + 1,130] = 76%). If 348 patients in the no-antibiotic group who experienced wound infection were replaced with hypothetical patients experiencing the population success rate (76%), then 76%×348 = 265 fewer patients would experience infection, leaving only 168 infections in that group. Study A would become Study A’, with no statistically significant difference between groups.

The surgeon can compare RIR = 348 against total sample size (*n* = 2,455) and ask whether, if the trial were repeated under different circumstances, it is likely for 348/2,455 = 14% of patients to experience this difference in wound infection probability. Because the RIR is adjusted for the outcome rate in the study population, it can also be compared across outcomes and studies, grounding debates about evidence strength in quantitative terms.

The RIR can also measure the robustness of a finding of *no* statistically significant difference: it calculates how many replacements would be required to produce a significant difference, with higher values indicating more robust evidence of no difference.

For continuous outcomes such as VAS pain, if MIS showed better post-operative pain control than open surgery, the RIR represents how many MIS patients would need to be replaced with hypothetical patients experiencing the average pain of open surgery patients for the result to lose significance. For continuous outcomes, we report the “fraction of the effect above/below the significance threshold” (FEAST/FEBST), interpretable as the percentage of the effect size that would need to be removed or added for the result to change.

It is important to note that there is no universally accepted consensus for what constitutes a “high”/“strong” versus “low”/“weak” RIR, FEAST/FEBST, or fragility. Interpretation relies on clinicians’ subjective estimations of whether the specified number of outcome changes is likely to occur in a hypothetical trial, and this interpretation relies on the study design and outcome measurement within the specific clinical context, which cannot be assessed in purely statistical terms. However, the goal of the RIR is not to definitively determine that any evidence is strong or weak. Instead, a richer use of the tool is to compare relative RIRs across outcomes, to determine which of the many conflicting conclusions made throughout the hernia literature are stronger or weaker. This is made possible by the fact that the RIR is measured on an intuitive scale based on patient event counts. Nevertheless, further methodological work will evaluate the RIR for outcomes that surgeons generally accept as true, such as the finding that prophylactic antibiotics reduce wound infections, to help establish relative thresholds for comparison.

Further methodological detail is in the Online Resource 1 Technical Appendix; theoretical detail about the RIR is in Frank et al., 2021 [2].

### Cumulative RIR analysis

**Fig. 3:**
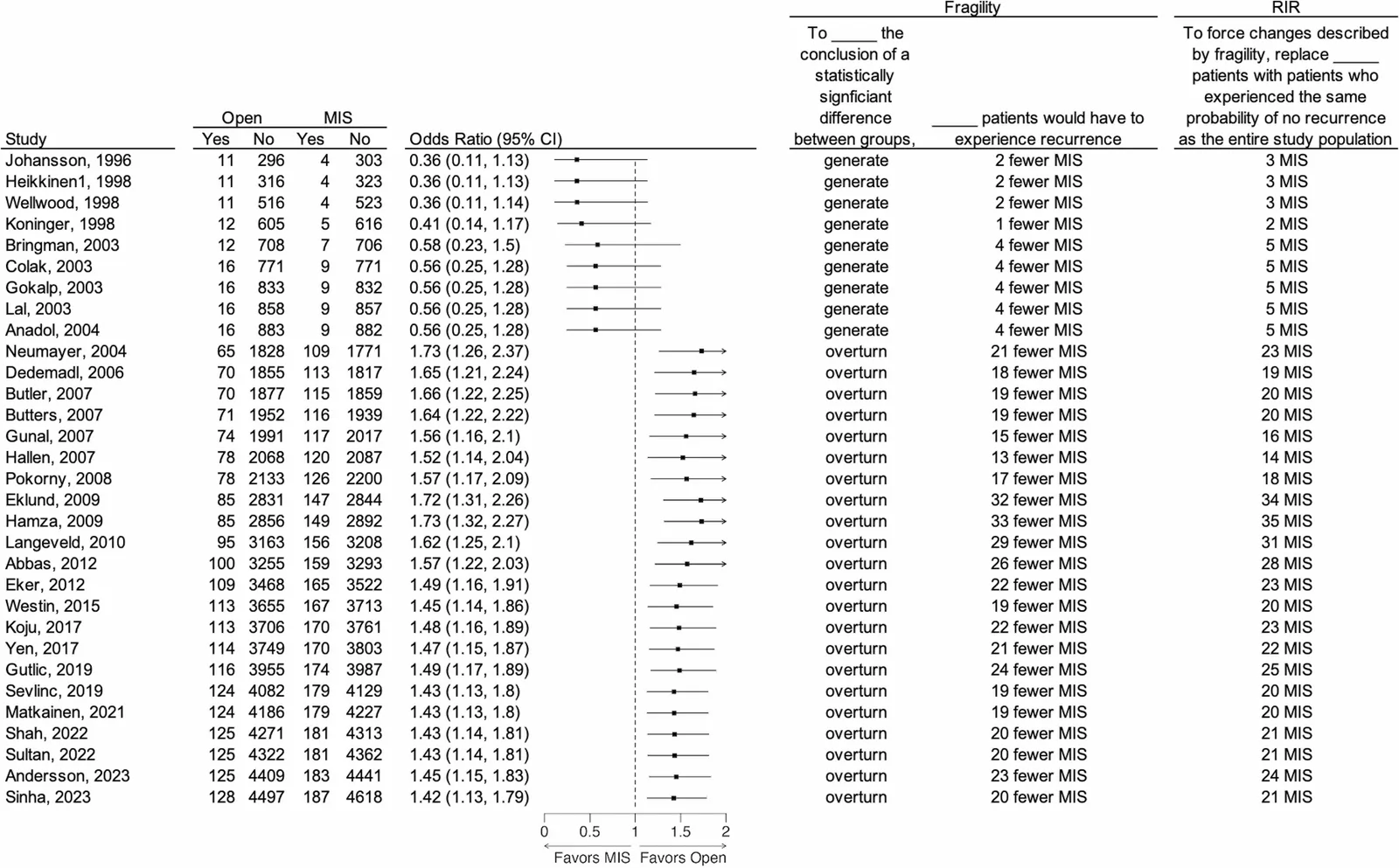
Cumulative sensitivity analysis for hernia recurrence after minimally invasive versus open surgery for unilateral elective inguinal hernia repair. NOTES: RIR = robustness of inference to replacement. MIS = minimally invasive surgery

**Fig. 4:**
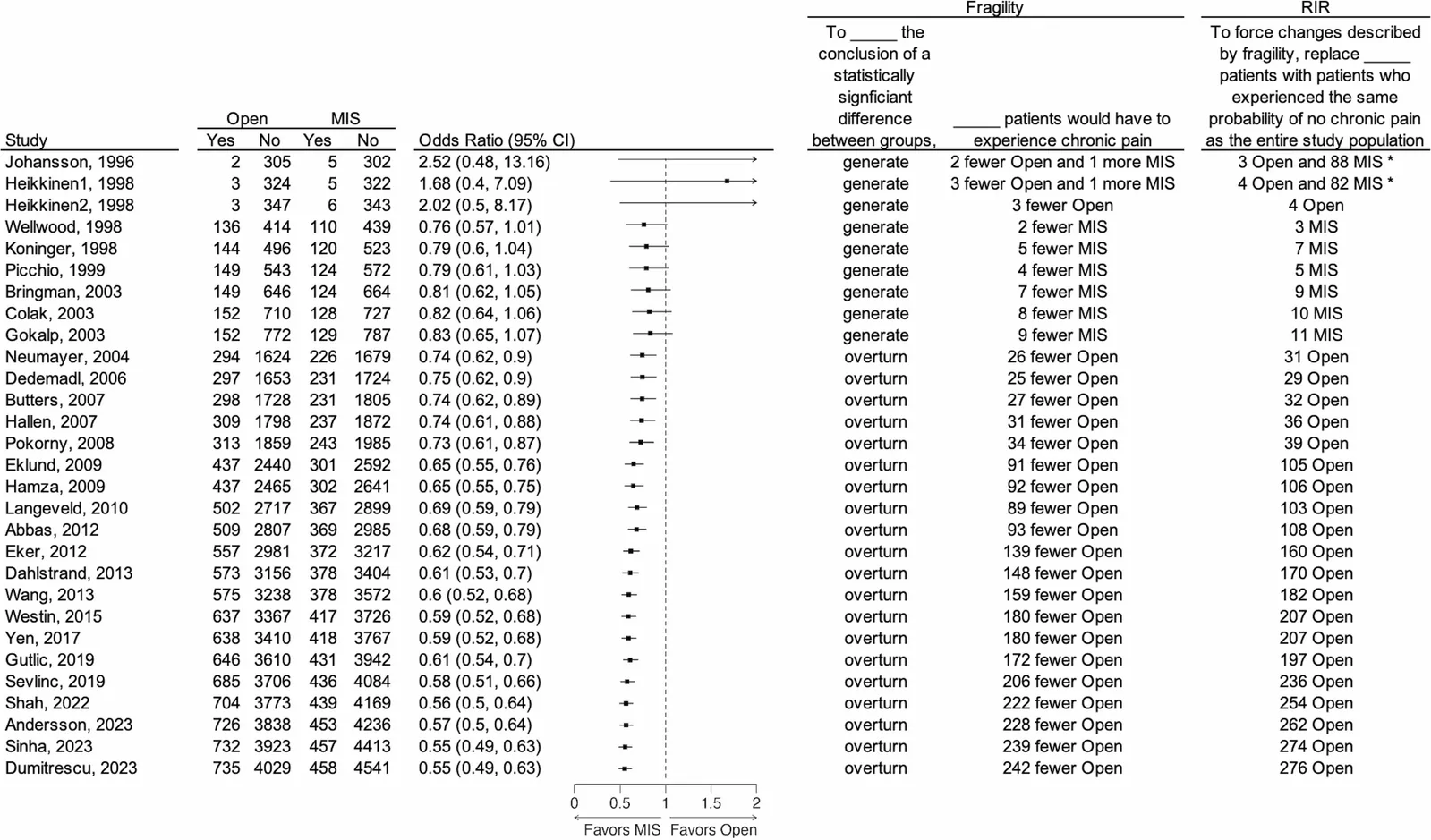
Cumulative sensitivity analysis for chronic pain after minimally invasive versus open surgery for unilateral elective inguinal hernia repair. NOTES: RIR = robustness of inference to replacement. MIS = minimally invasive surgery. *For more MIS patients to experience recurrence, the MIS patients in the published study need to be replaced with patients who experienced the same probability of experiencing chronic pain as the entire study population (versus not experiencing chronic pain as was the case for the other replacements.)

**Fig. 5:**
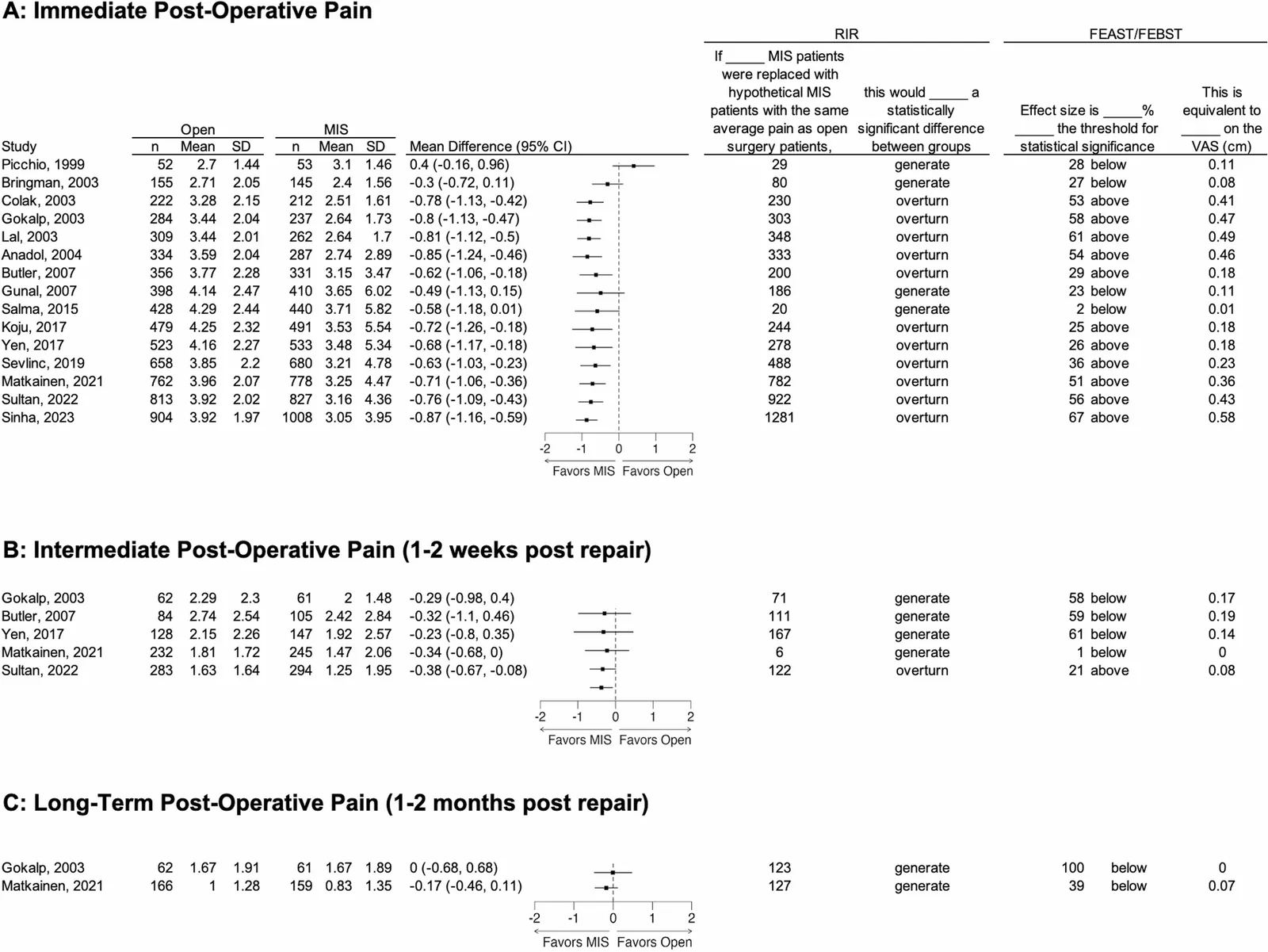
Cumulative Sensitivity Analysis for Post-Operative Pain measured on the Visual Analog Scale after Minimally Invasive versus Open Surgery for Unilateral Elective Inguinal Hernia Repair. NOTES: RIR = robustness of inference to replacement. MIS = minimally invasive surgery. FEAST = fraction of the effect above the significance threshold. FEBST = fraction of the effect below the significance threshold. VAS = Visual Analog Scale

After calculating the RIR for each outcome, we conducted a cumulative sensitivity analysis by updating RIR and Fragility sequentially as each new study was published. For dichotomous outcomes, complication counts from each new study were added to cumulative prior counts. For continuous outcomes, we calculated updated cumulative mean differences and standard deviations using a weighted average of effect sizes [2]. Results for hernia recurrence, chronic pain, and post-operative pain are presented in Figs. 3, 4 and 5; remaining dichotomous outcomes in Online Resource 1 Figs. 6, 7, 8 and 9; individual study results in Online Resource 1 Figs. 10, 11, 12, 13, 14, 15 and 16.

Analyses were conducted in R (version 4.5.1) using the publicly available package *konfound*.

### Sensitivity analysis limited to most recent data

Technical skills for MIS and patient selection for MIS versus open surgery have improved over time and may affect the risk of recurrence [6, 14]. We did not account for this in our primary analysis, since the goal was to describe how all the available evidence accumulated over time. However, to address these concerns, we conducted a sensitivity analysis where we restricted the analysis to studies published within the past 10 years (2016 or later), to focus on papers in which improved MIS technique may have substantially lowered the risk of recurrence.

## Results

### Literature review

Of 75 studies reviewed, 40 met inclusion criteria (Fig. 1), comprising 10,409 patients (5,165 MIS; 5,244 open surgery). Study characteristics are in Table 1; references in Online Resource 1 Table 2.

**Table 1:** Characteristics of included studies

Author and Year	Number of patients in each study arm				Follow-up duration	Outcomes Measured in each Study							
						Hernia Recurrence	Chronic Pain	Wound Infection	Hematoma/Seroma	Urinary Retention	Overall Morbidity	Immediate post-operative pain	Long-term post-operative pain
	Open	TAPP	TEP	Total MIS									
Johansson, 1996	307	307		307	12mo	X	X	X	X	X	X		
Heikkinen, 1998 (1)	20	20		20	median 7mo	X	X		X		X		
Heikkinen, 1998 (2)	23		22	22	1-2mo		X		X	X			
Wellwood, 1998	200	200		200	3mo	X	X	X	X	X			
Koninger, 1998	90	94			12mo	X	X	X	X				
Picchio, 1999	52	53		53	4wk		X		X		X	X	
Bringman, 2003	103		92	92	mean 19.8mo (SD 8.6mo)	X	X	X	X	X	X	X	
Colak, 2003	67		67	67	mean 12.84mo (SD2.84) mo for TEP, 11.10 SD 2.67 for open	X	X	X	X			X	
Gokalp, 2003	62		61	61	median 18mo, max 24mo	X	X	X	X	X	X	X	X
Lal, 2003	25		25	25	median 13mo (range 9-18mos)	X		X	X			X	
Anadol, 2004	25	25		25	avg 13.5mo (range 8-25mo)	X		X	X		X	X	
Neumayer, 2004	994		989	989	2yr	X	X		X	X	X		
Dedemadl, 2006	32	24	26	50	mean 1,087+/-588d	X	X		X	X	X		
Butler, 2007	22	22	22	44	patients seen in 7d intervals until they returned to work (mean 12 days)	X		X				X	
Butters, 2007	76	81		81	median 52mo (range 46-60mo)	X	X						
Gunal, 2007	42	39	40	79	mean 98±0.8 (open), 88±2.8 (TAPP), 87.2±1.1 (TEP)	X			X	X	X	X	
Hallen, 2007	81		73	73	median 7.3yr (range 6.1-8.9)	X	X						
Pokorny, 2008	65	85	34	119	3yr	X	X	X	X	X			
Eklund, 2009	705		665	665	5yr	X	X						
Hamza, 2009	25	25	25	50	24wk	X	X	X	X		X		
Langeveld, 2010	317		323	323	6wk, 1yr, 5yr (mean 49mo)	X	X	X	X	X	X		
Abbas, 2012	97	88		88	median 17.9 mo	X	X	X	X				
Eker, 2012	222		235	235	5yr	X	X						
Mesci, 2012	25	25	25	50	not reported			X	X	X			
Dahlstrand, 2013	191		193	193	6wk		X						
Wang, 2013	84	84	84	168	3mo to 32mo, avg 16mos		X		X		X		
Dhankhar, 2014	30		29	29	3mo				X	X	X		
Salma, 2015	30	30		30	immediate postoperative period							X	
Westin, 2015	191		193	193	1yr	X	X						
Koju, 2017	51	51		51	12mo	X		X	X		X	X	
Sawarkar, 2017	75		75		1wk, 1mo, 6mo			X	X	X	X		
Yen, 2017	44		42		6mo	X	X	X	X			X	
Gutlic, 2019	208		188	188	3yr	X	X	X	X		X		
Sevlinc, 2019	135		147	147	mean 40.9mo	X	X	X	X	X	X	X	
Matkainen, 2021	104		98	98	1mo	X		X	X		X	X	X
Shah, 2022	86		88	88	6mo	X	X	X	X	X			
Sultan, 2022	51	49			6mo	X		X	X		X	X	
Andersson, 2023	87		81	81	median 10 mo	X	X	X	X	X	X		
Sinha, 2023	91	181		181	3yr	X	X		X			X	
Dumitrescu, 2023	109	129		238	1yr		X	X	X				

### Hernia recurrence

Across all included studies, MIS was associated with increased odds of hernia recurrence. After the final study (Sinha et al., 2023), the cumulative odds ratio was 1.42 (95% CI 1.13, 1.79; Fig. 3). Cumulative Fragility was 20, meaning that if 20 fewer MIS patients experienced hernia recurrence there would be no statistically significant advantage to open surgery. Cumulative RIR was 21, meaning that to generate those 20 changes, 21 MIS patients who experienced recurrence would need to be replaced with hypothetical patients with the same cumulative probability of non-recurrence (97%) as the patients in all the included studies. Because recurrences were rare and non-recurrence probability was nearly 100%, the RIR and Fragility had similar magnitudes.

When early studies were published (1996–2003), there was a small but non-significant decrease in recurrence favoring MIS, but RIR values of 2–5 indicated that estimates were unstable and likely to change with future studies. Following Neumayer et al. (2004), the cumulative analysis showed open surgery led to fewer recurrences, and this conclusion was largely unchanged by subsequent studies. The RIR remained stable (23 after Neumayer; 21 after all studies), indicating that papers published after Neumayer et al. did not substantively change the conclusion or its robustness.

### Chronic pain

Across all included studies, MIS patients were less likely to experience chronic pain than open surgery patients (cumulative odds ratio 0.55, 95% CI 0.49, 0.63; Fig. 4). Cumulative Fragility was 242 and cumulative RIR was 276. From 1996 through Neumayer’s study (2004), the cumulative analysis found no significant difference in chronic pain by repair type. After 2004, however, the cumulative case counts demonstrated lower chronic pain after MIS, and the evidence became stronger with additional publications.

### Post-operative pain on the VAS

Across all included studies, MIS patients had less immediate post-operative pain than open surgery patients (mean difference − 0.87 cm, 95% CI -1.15, -0.59; Fig. 5). The cumulative RIR was 1,282, indicating that 1,282 of the 1,912 patients across the published literature would need to be replaced with patients experiencing no difference in pain score for the result to lose significance. The FEAST was 67%, equivalent to -0.58 cm on the VAS. For pain 1–2 weeks post-repair, MIS had less pain than open surgery (cumulative mean difference − 0.38 cm, 95% CI -0.68,-0.08), with cumulative RIR = 122 and FEAST = 21%, equivalent to -0.08 cm on the VAS. For pain 1–2 months post-repair, there was no significant difference; cumulative RIR was 127, equivalent to 0.07 cm on the VAS.

### Other outcomes

Results for the remaining outcomes are presented in Online Resource 1 Figs. 6, 7, 8 and 9. MIS had lower wound infection rates than open surgery (odds ratio 0.25, 95% CI 0.16, 0.40; cumulative RIR = 54) and increased urinary retention risk (odds ratio 1.51, 95% CI 1.06, 2.15; cumulative RIR = 5). There was no significant difference in hematoma/seroma (RIR = 38) or overall morbidity (RIR = 64).

### Sensitivity analysis limited to recent data

In the sensitivity analysis limited to data from the last 10 years, there was no significant difference in recurrence rate between open surgery and MIS (cumulative OR 1.24, 95% CI 0.63, 2.44; Online Resource 1 Table 3). The RIR for this estimate was 9, meaning that to generate a significant difference in the recurrence rate between MIS and open surgery, 9 open surgery patients would need to be replaced with patients who experienced the same probability of not experiencing the outcome as the entire study population. Similar to the primary analysis, MIS had lower incidence of chronic pain (cumulative OR 0.34, 95% CI 0.23, 0.50; RIR 49) and wound infection (cumulative OR 0.09, 95% CI 0.03, 0.24; RIR 34). There were no significant differences in hematoma/seroma or urinary retention between groups (RIRs 2 and 9, respectively). MIS had significantly less overall morbidity (RIR 10). The results for immediate, intermediate, and long-term post-operative pain were similar to the primary analysis.

## Discussion

Across all published studies, the evidence favoring MIS for reduced post-operative and chronic pain was substantially stronger than the evidence favoring open surgery for reduced hernia recurrence. The cumulative RIR for chronic pain (276) was more than 10 times larger than for recurrence (21). Although the RIR analysis across all post-operative outcomes did not definitively favor one approach over another, the analysis can inform better understanding of the evidence for outcomes that may matter differently to different patients. The RIR can also support more reasoned, quantitatively grounded arguments between disagreeing surgeons. For example, a surgeon who accepts RIR = 21 as sufficient to conclude MIS causes more recurrences should accept that MIS even more likely causes less chronic pain. The findings can also support guideline development grounded in a more nuanced interpretation of the evidence, guided by the question of whether additional studies are likely to change the overall conclusions.

Our sensitivity analysis limited to data from the past 10 years highlights how improving technical skills with time may have affected our results. This was especially apparent for hernia recurrence; when we eliminated the older studies, which tended to favor open surgery, there was no longer a statistically significant difference in recurrence between open surgery and MIS. However, the RIR for this outcome was relatively small (9) and still much smaller than the cumulative RIR in the limited sample for chronic pain (49). Surgeons could use these findings to argue MIS is superior to open surgery, but this claim may be weaker because it is based on a much smaller subset of the data. Importantly, the restriction to more recent studies eliminated the trial by Neumayer et al. [3], which was the single largest and best-designed study. It is unclear whether the changes in the findings were due to improvements in MIS or the elimination of an important study that favors open surgery. We do note there are other factors that may have affected the results, such as differences in outcomes when studies are conducted in specialized centers versus the general population, length of follow-up time, and differing practice patterns across different parts of the world [14]. While we did not directly assess these factors in this study, further work can explore how accounting for these factors affects the strength of the evidence in favor of either surgical approach for all the relevant post-operative outcomes.

Our study is one of the first applications of the RIR to appraising the totality of published evidence on a controversial surgical question [15–17]. The RIR enables reasoned arguments between surgeons who do and do not accept published findings. Rather than relying on subjective beliefs that any given study is “good” or “bad,” surgeons can quantify the relative robustness of evidence for different outcomes across studies and ask: if another study were performed comparing recurrence after MIS versus open surgery, how likely is it to change the overall conclusion? If a trial were repeated under different circumstances, how likely would it reach a different conclusion? If a surgeon shifts part of their hernia practice to MIS, how many fewer patients would experience chronic pain, and would this outweigh the burden of a higher recurrence rate requiring reoperation for a small number of patients?

The RIR provides a better understanding of the robustness of published findings than traditional statistics such as confidence intervals and p-values, which have crucial limitations and are difficult to conceptualize in terms of actual patient numbers [11]. The cumulative RIR also has an important advantage over traditional meta-analysis: it directly assesses the likelihood that an additional study would reverse the overall conclusion, helping surgeons decide whether existing evidence is strong enough to dictate practice. Unlike Fragility alone, the RIR accounts for the baseline rate of the outcome in the study population, enabling comparisons across different outcomes and populations [9]. Our chronic pain findings illustrated this point: the RIR was substantially higher than the Fragility, because the RIR captured not only what would need to change to generate a different result, but also how difficult it would be to generate that change given the incidence of the outcome. The RIR can also be applied when studies find no significant differences between groups, an additional advantage over Fragility [2].

Our study had limitations. First, efforts to quantify differences in pain were complicated by studies using different definitions of chronic pain and measuring it at different time points (Online Resource 1 Table 2). Second, in the cumulative sensitivity analysis, evidence from early years is weighted equally with evidence from later years, when there were likely technical improvements in MIS; however, change in technical skill is difficult to quantify, and we prioritized including all available evidence. Lastly, we did not differentiate between laparoscopic and robotic-assisted approaches, or between TAPP and TEP approaches, which is an important area for future work.

## Conclusion

The evidence supporting MIS for decreased post-operative and chronic pain is substantially stronger than the evidence supporting open surgery for decreased hernia recurrence. However, there is likely no clearly superior method for all patients. Our findings demonstrate how the RIR can support better surgical decision-making by helping surgeons interpret a large and complex body of published literature to make better and more personalized treatment decisions for their patients with inguinal hernias. 

## Electronic Supplementary Material

Below is the link to the electronic supplementary material.

Supplementary File 1 (DOCX 3.48 MB)
