# Supplementary material for: Outcomes after inguinal hernia repair: how strong is the evidence for minimally invasive versus open repair?
Source: Hernia. 2026 Sep 5;30(1):344. doi: 10.1007/s10029-026-03824-2 (PMC13546352; doi:10.1007/s10029-026-03824-2)
Supplement: Supplementary file 1 [file 10029_2026_3824_MOESM1_ESM.docx]

**Online Resource 1**

**Table 2: References for Included Studies and Varying Definitions of Chronic Pain**

| **Author, Year, and Reference** | **Study’s definition of chronic pain** |
| --- | --- |
|  |  |
|  |  |
| Johansson, 1996^1^ | pain/tenderness |
| Heikkinen1, 1998^2^ | pain for >1 month |
| Heikkinen2, 1998^3^ | ileoinguinal neuralgia |
| Wellwood, 1998^4^ | groin or thigh pain |
| Koninger, 1998^5^ | Beschwerden (article in German) |
| Picchio, 1999^6^ | groin discomfort or pain |
| Bringman, 2003^7^ | no definition provided |
| Colak, 2003^8^ | groin discomfort/pain |
| Gokalp, 2003^9^ | persistent pain/tenderness |
| Lal, 2003^10^ | no definition provided |
| Anadol, 2004^11^ | no definition provided |
| Neumayer, 2004^12^ | neuralgia or other pain |
| Dedemadl, 2006^13^ | neuralgia or testicular pain |
| Butler, 2007^14^ | no definition provided |
| Butters, 2007^15^ | lateral cutaneous nerve damage |
| Gunal, 2007^16^ | no definition provided |
| Hallen, 2007^17^ | chronic groin pain |
| Pokorny, 2008^18^ | persistent pain/tenderness |
| Eklund, 2009^19^ | discomfort that occasionally interferes with daily activities, or more severe |
| Hamza, 2009^20^ | groin pain |
| Langeveld, 2010^21^ | pain/sensibility after 1 year |
| Abbas, 2012^22^ | pain lasting 3 months or more |
| Eker, 2012^23^ | sensibility disorder or numbness |
| Mesci, 2012^24^ | no definition provided |
| Dahlstrand, 2013^25^ | pain in the operated groin interfering with concentration during daily activities |
| Wang, 2013 | no definition provided |
| Dhankhar, 2014^27^ | no definition provided |
| Salma, 2015^28^ | no definition provided |
| Westin, 2015^29^ | persistent pain |
| Koju, 2017^30^ | no definition provided |
| Sawarkar, 2017^31^ | no definition provided |
| Yen, 2017^32^ | groin pain at 6 months |
| Gutlic, 2019^33^ | non-ignorable pain in the last week |
| Sevlinc, 2019^34^ | pain lasting more than 3 months after surgery |
| Matkainen, 2021^35^ | no definition provided |
| Shah, 2022^36^ | presence of postoperative pain |
| Sultan, 2022^37^ | no definition provided |
| Andersson, 2023^38^ | no definition provided |
| Sinha, 2023^39^ | chronic post operative inguinal pain persisting for 3 or more months |
| Dumitrescu, 2023^40^ | postoperative inguinodynia |

**References**

1. Johansson B, Hallerbäck B, Glise H, Anesten B, Smedberg S, Román J (1999) Laparoscopic Mesh Versus Open Preperitoneal Mesh Versus Conventional Technique for Inguinal Hernia Repair. Ann Surg. 230(2):225.
2. Heikkinen TJ, Haukipuro K, Koivukangas P, Hulkko A (1998) A prospective randomized outcome and cost comparison of totally extraperitoneal endoscopic hernioplasty versus Lichtenstein hernia operation among employed patients. Surg Laparosc Endosc. 8(5):338-344.
3. Heikkinen TJ, Haukipuro K, Hulkko A (1998) A cost and outcome comparison between laparoscopic and Lichtenstein hernia operations in a day-case unit. A randomized prospective study. Surg Endosc. 12(10):1199-1203. doi:10.1007/s004649900820
4. Wellwood J, Sculpher MJ, Stoker D, et al (1998) Randomised controlled trial of laparoscopic versus open mesh repair for inguinal hernia: outcome and cost. BMJ. 317(7151):103-110. doi:10.1136/bmj.317.7151.103
5. Köninger JS, Oster M, Butters M (1998) [Management of inguinal hernia--a comparison of current methods]. Chir Z Alle Geb Oper Medizen. 69(12):1340-1344. doi:10.1007/s001040050581
6. Picchio M, Lombardi A, Zolovkins A, Mihelsons M, La Torre G (1999) Tension-free laparoscopic and open hernia repair: randomized controlled trial of early results. World J Surg. 23(10):1004-1007; discussion 1008-1009. doi:10.1007/s002689900615
7. Bringman S, Ramel S, Heikkinen TJ, Englund T, Westman B, Anderberg B (2003) Tension-Free Inguinal Hernia Repair: TEP Versus Mesh-Plug Versus Lichtenstein. Ann Surg. 237(1):142-147.
8. Colak T, Akca T, Kanik A, Aydin S (2003) Randomized clinical trial comparing laparoscopic totally extraperitoneal approach with open mesh repair in inguinal hernia. Surg Laparosc Endosc Percutan Tech. 13(3):191-195. doi:10.1097/00129689-200306000-00010
9. Gokalp A, Inal M, Maralcan G, Baskonus I (2003) A prospective randomized study of Lichtenstein open tension-free versus laparoscopic totally extraperitoneal techniques for inguinal hernia repair. Acta Chir Belg. 103(5):502-506. doi:10.1080/00015458.2003.11679476
10. Lal P, Kajla RK, Chander J, Saha R, Ramteke VK (2003) Randomized controlled study of laparoscopic total extraperitoneal versus open Lichtenstein inguinal hernia repair. Surg Endosc. 17(6):850-856. doi:10.1007/s00464-002-8575-6
11. Anadol ZA, Ersoy E, Taneri F, Tekin E (2004) Outcome and cost comparison of laparoscopic transabdominal preperitoneal hernia repair versus Open Lichtenstein technique. J Laparoendosc Adv Surg Tech A. 14(3):159-163. doi:10.1089/1092642041255414
12. Neumayer L, Giobbie-Hurder A, Jonasson O, et al (2004) Open mesh versus laparoscopic mesh repair of inguinal hernia. N Engl J Med. 350(18):1819-1827. doi:10.1056/NEJMoa040093
13. Dedemadi G, Sgourakis G, Karaliotas C, Christofides T, Kouraklis G, Karaliotas C (2006) Comparison of laparoscopic and open tension-free repair of recurrent inguinal hernias: a prospective randomized study. Surg Endosc. 20(7):1099-1104. doi:10.1007/s00464-005-0621-8
14. Butler RE, Burke R, Schneider JJ, Brar H, Lucha PA (2007) The economic impact of laparoscopic inguinal hernia repair: results of a double-blinded, prospective, randomized trial. Surg Endosc. 21(3):387-390. doi:10.1007/s00464-006-9123-6
15. Butters M, Redecke J, Köninger J (2007) Long-term results of a randomized clinical trial of Shouldice, Lichtenstein and transabdominal preperitoneal hernia repairs. Br J Surg. 94(5):562-565. doi:10.1002/bjs.5733
16. Günal O, Ozer S, Gürleyik E, Bahçebaşi T (2007) Does the approach to the groin make a difference in hernia repair? Hernia J Hernias Abdom Wall Surg. 11(5):429-434. doi:10.1007/s10029-007-0252-1
17. Hallén M, Bergenfelz A, Westerdahl J (2008) Laparoscopic extraperitoneal inguinal hernia repair versus open mesh repair: long-term follow-up of a randomized controlled trial. Surgery. 143(3):313-317. doi:10.1016/j.surg.2007.09.028
18. Pokorny H, Klingler A, Schmid T, et al (2008) Recurrence and complications after laparoscopic versus open inguinal hernia repair: results of a prospective randomized multicenter trial. Hernia J Hernias Abdom Wall Surg. 12(4):385-389. doi:10.1007/s10029-008-0357-1
19. Eklund AS, Montgomery AK, Rasmussen IC, Sandbue RP, Bergkvist LA, Rudberg CR (2009) Low recurrence rate after laparoscopic (TEP) and open (Lichtenstein) inguinal hernia repair: a randomized, multicenter trial with 5-year follow-up. Ann Surg. 249(1):33-38. doi:10.1097/SLA.0b013e31819255d0
20. Hamza Y, Gabr E, Hammadi H, Khalil R (2010) Four-arm randomized trial comparing laparoscopic and open hernia repairs. Int J Surg Lond Engl. 8(1):25-28. doi:10.1016/j.ijsu.2009.09.010
21. Langeveld HR, van't Riet M, Weidema WF, et al (2010) Total extraperitoneal inguinal hernia repair compared with Lichtenstein (the LEVEL-Trial): a randomized controlled trial. Ann Surg. 251(5):819-824. doi:10.1097/SLA.0b013e3181d96c32
22. Abbas AE, Abd Ellatif ME, Noaman N, et al (2012) Patient-perspective quality of life after laparoscopic and open hernia repair: a controlled randomized trial. Surg Endosc. 26(9):2465-2470. doi:10.1007/s00464-012-2212-9
23. Eker HH, Langeveld HR, Klitsie PJ, et al (2012) Randomized clinical trial of total extraperitoneal inguinal hernioplasty vs Lichtenstein repair: a long-term follow-up study. Arch Surg Chic Ill 1960. 147(3):256-260. doi:10.1001/archsurg.2011.2023
24. Mesci A, Korkmaz B, Dinckan A, Colak T, Balci N, Ogunc G (2012) Digital evaluation of the muscle functions of the lower extremities among inguinal hernia patients treated using three different surgical techniques: a prospective randomized study. Surg Today. 42(2):157-163. doi:10.1007/s00595-011-0017-4
25. Dahlstrand U, Sandblom G, Ljungdahl M, Wollert S, Gunnarsson U (2013) TEP under general anesthesia is superior to Lichtenstein under local anesthesia in terms of pain 6 weeks after surgery: results from a randomized clinical trial. Surg Endosc. 27(10):3632-3638. doi:10.1007/s00464-013-2936-1
26. Wang WJ, Chen JZ, Fang Q, Li JF, Jin PF, Li ZT (2013) Comparison of the effects of laparoscopic hernia repair and Lichtenstein tension-free hernia repair. J Laparoendosc Adv Surg Tech A. 23(4):301-305. doi:10.1089/lap.2012.0217
27. Dhankhar DS, Sharma N, Mishra T, Kaur N, Singh S, Gupta S (2014) Totally extraperitoneal repair under general anesthesia versus Lichtenstein repair under local anesthesia for unilateral inguinal hernia: a prospective randomized controlled trial. Surg Endosc. 28(3):996-1002. doi:10.1007/s00464-013-3269-9
28. Salma U, Ahmed I, Ishtiaq S (2015) A comparison of post operative pain and hospital stay between Lichtenstein's repair and Laparoscopic Transabdominal Preperitoneal (TAPP) repair of inguinal hernia: A randomized controlled trial. Pak J Med Sci. 31(5):1062-1066. doi:10.12669/pjms.315.4811
29. Westin L, Wollert S, Ljungdahl M, Sandblom G, Gunnarsson U, Dahlstrand U (2016) Less Pain 1 Year After Total Extra-peritoneal Repair Compared With Lichtenstein Using Local Anesthesia: Data From a Randomized Controlled Clinical Trial. Ann Surg. 263(2):240-243. doi:10.1097/SLA.0000000000001289
30. Koju R, Koju RB, Malla B, Dongol Y, Thapa LB (2017) Transabdominal Pre-peritoneal Mesh Repair versus Lichtenstein's Hernioplasty. J Nepal Health Res Counc. 15(2):135-140. doi:10.3126/jnhrc.v15i2.18202
31. Sawarkar P, Agrawal A, Zade R, Mehera B, Gupta D (2017) Lichtenstein Hernia Repair Versus Totally Extraperitoneal Hernia Repair: Randomized Control Study in Rural Area. J Mahatma Gandhi Inst Med Sci. 22(2):93. doi:10.4103/jmgims.jmgims_47_15
32. Yen FV (2013) Laparoscopic total extraperitoneal approach versus open lichtenstein repair of primary unilateral uncomplicated inguinal hernia: a prospective randomised controlled trial in Hospital Selayang, selangor. Accessed March 9, 2026. <https://erepo.usm.my/handle/123456789/19543>
33. Gutlic N, Gutlic A, Petersson U, Rogmark P, Montgomery A (2019) Randomized clinical trial comparing total extraperitoneal with Lichtenstein inguinal hernia repair (TEPLICH trial). Br J Surg. 106(7):845-855. doi:10.1002/bjs.11230
34. Sevinç B, Damburacı N, Güner M, Karahan Ö (2019) Comparison of early and long term outcomes of open Lichtenstein repair and totally extraperitoneal herniorrhaphy for primary inguinal hernias. Turk J Med Sci. 49(1):38-41. doi:10.3906/sag-1803-94
35. Matikainen M, Vironen JH, Silvasti S, et al (2021) A randomized clinical trial comparing early patient-reported pain after open anterior mesh repair versus totally extraperitoneal repair of inguinal hernia. Br J Surg. 108(12):1433-1437. doi:10.1093/bjs/znab354
36. Shah MY, Raut P, Wilkinson TRV, Agrawal V (2022) Surgical outcomes of laparoscopic total extraperitoneal (TEP) inguinal hernia repair compared with Lichtenstein tension-free open mesh inguinal hernia repair: A prospective randomized study. Medicine (Baltimore). 101(26):e29746. doi:10.1097/MD.0000000000029746
37. Sultan AAEA, Abo Elazm HA, Omran H (2022) Lichtenstein versus transabdominal preperitoneal (TAPP) inguinal hernia repair for unilateral non recurrent hernia: A multicenter short term randomized comparative study of clinical outcomes. Ann Med Surg. 76:103428. doi:10.1016/j.amsu.2022.103428
38. Andersson B, Hallén M, Leveau P, Bergenfelz A, Westerdahl J (2003) Laparoscopic extraperitoneal inguinal hernia repair versus open mesh repair: a prospective randomized controlled trial. Surgery. 133(5):464-472. doi:10.1067/msy.2003.98
39. Sinha R, Yadav AS, Sharma Y, Chanda S, Sharma OK, Srivastava N (2023) Modified Open Anterior Preperitoneal Repair. JSLS J Soc Laparosc Robot Surg. 27(4):e2023.00044. doi:10.4293/JSLS.2023.00044
40. Dumitrescu V, Serban D, Costea DO, et al (2023) Transabdominal Preperitoneal Versus Lichtenstein Procedure for Inguinal Hernia Repair in Adults: A Comparative Evaluation of the Early Postoperative Pain and Outcomes. Cureus. 15(7):e41886. doi:10.7759/cureus.41886

**Figure 6: Cumulative Sensitivity Analysis for Wound Infection after Minimally Invasive versus Open Surgery for Unilateral Elective Inguinal Hernia Repair**

NOTES: RIR = robustness of inference to replacement. MIS = minimally invasive surgery. *An RIR cannot be calculated for an odds ratio of 0.

**Figure 7: Cumulative Sensitivity Analysis for Hematoma/Seroma after Minimally Invasive versus Open Surgery for Unilateral Elective Inguinal Hernia Repair**

NOTES: RIR = robustness of inference to replacement. MIS = minimally invasive surgery.

**Figure 8: Cumulative Sensitivity Analysis for Urinary Retention after Minimally Invasive versus Open Surgery for Unilateral Elective Inguinal Hernia Repair**

NOTES: RIR = robustness of inference to replacement. MIS = minimally invasive surgery. *For the second set of replacements, the patients in the published study need to be replaced with patients who experienced the same probability of experiencing the outcome as the entire study population (versus *not* experiencing the outcome as was the case for the other replacements.)

**Figure 9: Cumulative Sensitivity Analysis for Overall Morbidity after Minimally Invasive versus Open Surgery for Unilateral Elective Inguinal Hernia Repair**

NOTES: RIR = robustness of inference to replacement. MIS = minimally invasive surgery.

**Figure 10: Individual study Fragility and RIR for Hernia Recurrence after Minimally Invasive versus Open Surgery for Unilateral Elective Inguinal Hernia Repair**

NOTES: RIR = robustness of inference to replacement. MIS = minimally invasive surgery. *For the second set of replacements, the patients in the published study need to be replaced with patients who experienced the same probability of experiencing the outcome as the entire study population (versus *not* experiencing the outcome as was the case for the other replacements.) **An RIR cannot be calculated when the odds ratio is zero or undefined.

**Figure 11: Individual study Fragility and RIR for Chronic Pain after Minimally Invasive versus Open Surgery for Unilateral Elective Inguinal Hernia Repair**

NOTES: RIR = robustness of inference to replacement. MIS = minimally invasive surgery. *For the second set of replacements, the patients in the published study need to be replaced with patients who experienced the same probability of experiencing the outcome as the entire study population (versus *not* experiencing the outcome as was the case for the other replacements.) **An RIR cannot be calculated when the odds ratio is zero or undefined.

**Figure 12: Individual study Fragility and RIR for Wound Infection after Minimally Invasive versus Open Surgery for Unilateral Elective Inguinal Hernia Repair**

NOTES: RIR = robustness of inference to replacement. MIS = minimally invasive surgery. *For the second set of replacements, the patients in the published study need to be replaced with patients who experienced the same probability of experiencing the outcome as the entire study population (versus *not* experiencing the outcome as was the case for the other replacements.) **An RIR cannot be calculated when the odds ratio is zero or undefined.

**Figure 13: Individual study Fragility and RIR for Hematoma/Seroma after Minimally Invasive versus Open Surgery for Unilateral Elective Inguinal Hernia Repair**

NOTES: RIR = robustness of inference to replacement. MIS = minimally invasive surgery. *For the second set of replacements, the patients in the published study need to be replaced with patients who experienced the same probability of experiencing the outcome as the entire study population (versus *not* experiencing the outcome as was the case for the other replacements.) **An RIR cannot be calculated when the odds ratio is zero or undefined.

**Figure 14: Individual study Fragility and RIR for Urinary Retention after Minimally Invasive versus Open Surgery for Unilateral Elective Inguinal Hernia Repair**

NOTES: RIR = robustness of inference to replacement. MIS = minimally invasive surgery. *For the second set of replacements, the patients in the published study need to be replaced with patients who experienced the same probability of experiencing the outcome as the entire study population (versus *not* experiencing the outcome as was the case for the other replacements.) **An RIR cannot be calculated when the odds ratio is zero or undefined.

**Figure 15: Individual study Fragility and RIR for Overall Morbidity after Minimally Invasive versus Open Surgery for Unilateral Elective Inguinal Hernia Repair**

NOTES: RIR = robustness of inference to replacement. MIS = minimally invasive surgery. *For the second set of replacements, the patients in the published study need to be replaced with patients who experienced the same probability of experiencing the outcome as the entire study population (versus *not* experiencing the outcome as was the case for the other replacements.) **An RIR cannot be calculated when the odds ratio is zero or undefined.

**Figure 16: RIR and FEAST/FEBST for Individual Studies Measuring Post-Operative Pain on the Visual Analog Scale after Minimally Invasive versus Open Surgery for Unilateral Elective Inguinal Hernia Repair**

NOTES: RIR = robustness of inference to replacement. MIS = minimally invasive surgery. FEAST = fraction of the effect above the significance threshold. FEBST = fraction of the effect below the significance threshold. VAS = Visual Analog Scale.

**Table 3: Sensitivity analysis limited to studies published in the past 10 years (2016 or later)**

A. **Dichotomous outcomes**

| **Outcome** | **Open** | | **MIS** | | **Cumulative odds ratio** | **Favored approach** | **Cumulative fragility**  (To ___ a conclusion of a statistically significant difference between groups; ___ patients must experience the outcome) | **Cumulative RIR**  (To force changes described by fragility, replace ___ patients with patients who experienced the same probability of not experiencing the outcome as the entire study population) |
| --- | --- | --- | --- | --- | --- | --- | --- | --- |
|  | **Yes** | **No** | **Yes** | **No** |  |  |  |  |
| Recurrence | 15 | 842 | 20 | 905 | 1.24 (0.63, 2.44) | Open, but not significant | Generate; 8 fewer open | 9 open |
| Chronic pain | 98 | 662 | 41 | 815 | 0.34 (0.23, 0.50) | MIS | Overturn; 44 fewer open | 49 open |
| Wound infection | 45 | 905 | 4 | 944 | 0.09 (0.03, 0.24) | MIS | Overturn; 33 fewer open | 34 open |
| Hematoma/seroma | 64 | 977 | 48 | 1081 | 0.68 (0.46, 1.00) | MIS, but not significant | Generate; 1 fewer MIS | 2 MIS |
| Urinary retention | 11 | 372 | 12 | 379 | 1.07 (0.47, 2.46) | Open, but not significant | Generate; 1 fewer open | 9 open |
| Overall morbidity | 63 | 648 | 35 | 654 | 0.55 (0.36, 0.84) | MIS | Overturn; 9 fewer open | 10 open |

B. **Continuous outcomes**

| **Outcome** | **Open** | | | **MIS** | | | **Cumulative effect size, VAS (cm)** | **Favored approach** | **Cumulative RIR**  (If ___ MIS patients were replaced with hypothetical MIS patients with the same average pain as open surgery patients; this would ___ a statistically significant difference between groups) | **Cumulative FEAST/FEBST**  (Effect size is ___% ___ the threshold for statistical significance. This is equivalent to ___ on the VAS (cm) |
| --- | --- | --- | --- | --- | --- | --- | --- | --- | --- | --- |
|  | **n** | **mean** | **SD** | **n** | **Mean** | **SD** |  |  |  |  |
| Immediate post-operative pain | 904 | 3.92 | 1.97 | 1008 | 3.05 | 3.95 | -0.87 (-1.16, -0.59) | MIS | 1281; overturn | 67% above; -0.58 cm |
| Intermediate post-operative pain | 283 | 1.63 | 1.64 | 294 | 1.25 | 1.95 | -0.38 (-0.67, -0.08) | MIS | 122; overturn | 21% above; -0.08 cm |
| Long-term post-operative pain | 166 | 1 | 1.28 | 159 | 0.83 | 1.35 | -0.17 (-0.46, 0.11) | MIS, but not significant | 127; generate | 39% below, -0.07 cm |

NOTES: RIR = robustness of inference to replacement. MIS = minimally invasive surgery. FEAST = fraction of the effect above the significance threshold. FEBST = fraction of the effect below the significance threshold. VAS = Visual Analog Scale.

**Technical Appendix**

Further discussion about the theory of the RIR is provided here: *Frank KA, Lin Q, Maroulis S, et al. Hypothetical case replacement can be used to quantify the robustness of trial results. J Clin Epidemiol. 2021;134:150-159. doi:10.1016/j.jclinepi.2021.01.025*

This appendix outlines the analytic decisions made by our team of authors while applying the RIR method, which was originally developed in the field of education, to a research question in general surgery.

RIR for dichotomous outcomes

For findings reported as odds ratios derived from standard epidemiological 2x2 tables, the RIR software asks the researcher to answer two specific questions:

1. For the calculation of Fragility: should case movement originate in the treatment group or the control group?
2. For the conversion from Fragility to RIR: should this use the probability of the outcome in the control group, or should this use the probability of the outcome in the entire study population?

The answers to these questions affect the RIR results. For the current study, we programmed the software as follows. We made these decisions with the motivation to allow the RIR to be comparable across different studies and outcomes.

1. When there **is** **a statistically significant difference** between MIS and open surgery, case movement should originate in the group with higher incidence of the outcome. When there **is no statistically significant difference**, case movement should originate in the group with lower incidence of the outcome.
   - This allows Fragility to be conceptualized in every scenario as how many fewer complications must occur for the result to change. This reflects the fact that many surgeons believe that technical advancements in both open surgery (e.g. targeted injection of local anesthetic and ERAS protocols) and MIS (e.g. improved laparoscopic skills) may result in fewer complications.
   - Therefore, the “destination” cell within the 2x2 table is always within the row for “success” (no complication). This is represented in the 2x2 table below. Regardless of whether the outcome occurs more frequently in the MIS or open surgery groups, or whether there is or is not a statistically significant different between groups, the “destination” cell for the case movement will always be C or D.

|  | Open Surgery | MIS |
| --- | --- | --- |
| Complication (“failure”) | A | B |
| No Complication (“success”) | C | D |

- Remember that the probability of patients landing in this “destination” cell is used to convert the Fragility into RIR [RIR = Fragility / P(destination cell)]. So, for this calculation, the “outcome” is always the probability of *success*, meaning *non-*occurrence of the complication, regardless of whether this is within the open surgery group or the MIS group. Importantly, this “destination cell” represents the “null hypothesis population” from which case replacements are drawn.
- If case movement were instead restricted to originate from either the open surgery group or the MIS group in every case, then sometimes the “destination” cell would be “success” or “failure” depending on what type of case movement is required to overturn the inference. This would lead to major inconsistencies in the RIR between studies favoring open surgery versus MIS, because of the large difference in magnitude between the probability of “failure” (usually close to 0) and the probability of “success” (usually close to 1) for the relatively rare study outcomes.
- *NOTE: This decision is appropriate for our scenario because MIS and open surgery are conceptually “equivalent” treatments. It does not really matter whether open surgery or MIS is specified as “treatment” or “control.” In other settings where there would be a more obvious treatment and control, such as comparing non-operative to operative management for acute appendicitis, it may make more sense to ensure that case replacements always begin in the control group [operative management], regardless of which group has the higher outcome rate. This would ensure that case replacements always reflect the concept of replacement from a “null hypothesis” population.*

2. To convert Fragility into RIR, always use the probability of the outcome in the entire study population. We made this decision to ensure consistency of the calculations across many different studies for many different outcomes with results that sometimes favored open surgery and sometimes favored MIS.

- *NOTE: in other scenarios, one may choose to always use the probability of the outcome in the control group to convert Fragility to RIR. The argument for this approach is that this always leads to a smaller RIR estimate and thus a more conservative conclusion about the strength of the evidence supporting the inference.*

The following diagram depicts how our answers to Questions 1 and 2 affect the directions of case transfer and probabilities used for case replacement in different situations:

The RIR software also allows the researcher to specify whether the Chi-squared test or Fisher’s exact test should be used to determine statistical significance. We chose Fisher’s exact test because this was more reliable when there were small cell sizes. There were many studies where very small numbers of patients in both the open surgery and MIS groups experienced the study outcomes.

There were several studies where case transfers within the study group with higher incidence of the outcome were not sufficient to overturn the inference and change the study result. This often happened when the difference in the incidence rate between the study groups was relatively large, and it was more likely to happen when the overall incidence rate was relatively low. In these situations, the RIR could still be calculated by initiating additional case transfers within the study group with lower incidence of the outcome. This is called a “double switch,” because overturning the association between repair type and outcome incidence would require both (1) in the group with higher outcome incidence, fewer patients experiencing the outcome, and (2) in the group with lower outcome incidence, more patients experiencing the outcome. In these cases, we reported Fragility and RIR as two separate numbers, indicating the case transfers and replacements required for both (1) and (2). For (2), note that the patients in the published study need to be replaced with hypothetical patients who experienced the same probability of experiencing the outcome as the entire study population (versus *not* experiencing the outcome as was the case for the other replacements.) Since the outcomes in our study were relatively rare, this resulted in higher RIRs, reflecting the fact that it would be difficult to identify these additional patients who experienced the outcomes of interest by sampling from the null hypothesis population.

There were some cases where calculating the RIR for a specific study was impossible. If zero patients in the Open Surgery group experienced the outcome, then the odds ratio for the association between MIS and the outcome was undefined, and therefore so too the RIR was undefined**.** (It is still possible to calculate an RIR for a study where zero MIS patients experience an outcome but one open surgery patient does experience the outcome.)

Note about our approach to the cumulative analysis

As the results of each “new” study were added to the cumulative case counts, when we calculated Fragility, we allowed the directionality of the case transfers to change if there was a change in which study group (either MIS or open surgery) had higher incidence of the outcome. This helped ensure that in every row of the figure, a probability of “success” was always used to convert Fragility to RIR, to support consistency in interpretation the RIR.

Alternatively, in each row of the figure, we could have forced the directionality of Fragility to match the direction indicated at the final time point in the cumulative analysis, after the last study was added. This would have ensured consistency in interpretation of Fragility across each row of the figure, in the spirit of traditional meta-analysis, but it does not reflect the state of the data at earlier time points before the final outcome is known, avoiding presentism bias.

RIR for continuous outcomes

For continuous outcomes, the RIR is designed to evaluate the strength of an inference described by a mean difference and its 95% confidence interval.

Most studies reported the average pain scores in the open surgery and MIS groups and provided p-values to assess statistical significance. We used standard statistical formulae to convert the p-values into standard errors, to calculate the mean difference between groups, and then to calculate the 95% confidence interval for this mean difference using the pooled standard error. Studies were only included when they provided the mean and standard deviation (or standard error) for both open surgery patients and MIS patients. When a study reported only median and interquartile range, we could not include it in our analysis.
